# Dynamic Addressing Molecular Robot (DAMR): An Effective and Efficient Trial‐and‐Error Approach for the Analysis of Single Nucleotide Polymorphisms

**DOI:** 10.1002/advs.202402140

**Published:** 2024-06-17

**Authors:** Tianshu Chen, Guifang Chen, Siyu Cao, Xiaochen Tang, Wenxing Li, Chenbin Liu, Hongquan Gou, Pei Sun, Yichun Mao, Qiuhui Pan, Penghui Zhang, Xiaoli Zhu

**Affiliations:** ^1^ Clinical Laboratory, Shanghai Children's Medical Center School of Medicine Shanghai Jiao Tong University Shanghai 200127 P. R. China; ^2^ Shanghai Key Laboratory of Clinical Molecular Diagnostics for Pediatrics Shanghai 200127 P. R. China; ^3^ Center for Molecular Recognition and Biosensing, School of Life Sciences Shanghai University Shanghai 200444 P. R. China; ^4^ Department of Clinical Laboratory Medicine Shanghai Tenth People's Hospital of Tongji University Shanghai 200072 P. R. China; ^5^ Department of Laboratory Medicine Shanghai Pudong New Area People's Hospital Shanghai 201299 P. R. China

**Keywords:** DNA framework, microRNA, molecular recognition, molecular robot, single nucleotide polymorphism

## Abstract

Accurate and efficient molecular recognition plays a crucial role in the fields of molecular detection and diagnostics. Conventional trial‐and‐error‐based molecular recognition approaches have always been challenged in distinguishing minimal differences between targets and non‐targets, such as single nucleotide polymorphisms (SNPs) of oligonucleotides. To address these challenges, here, a novel concept of dynamic addressing analysis is proposed. In this concept, by dissecting the regions of the target and creating a corresponding recognizer, it is possible to eliminate the inaccuracy and inefficiency of recognition. To achieve this concept, a Dynamic Addressing Molecular Robot (DAMR), a DNA‐based dynamic addressing device is developed which is capable of dynamically locating targets. DAMR is designed to first bind to the conserved region of the target while addressing the specific region dynamically until accurate recognition is achieved. DAMR has provided an approach for analyzing low‐resolution targets and has been used for analyzing SNP of miR‐196a2 in both cell and serum samples, which has opened new avenues for effective and efficient molecular recognition.

## Graphical abstract

This work proposes a novel concept of dynamic addressing analysis by designing a Dynamic Addressing Molecular Robot to accurately and efficiently distinguish multiple targets that have minimal distinctions in complex systems. This method shows higher accuracy and efficiency compared to traditional methods, demonstrating good analysis performance in both cells and serum samples.

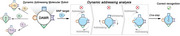

## 1 Introduction

**Figure 1:**
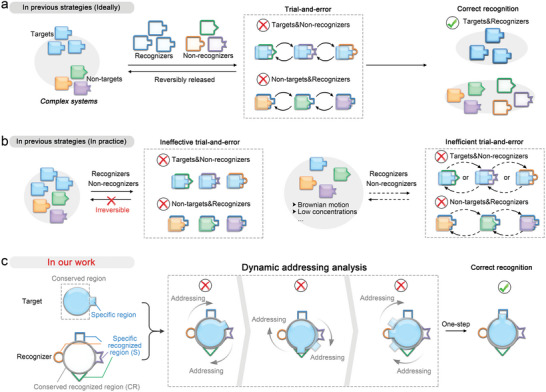
Comparison of target analysis by previous trial‐and‐error or dynamic addressing analysis. a) Schematic illustration of target analysis by previous trial‐and‐error strategies in the ideal case. b) Schematic illustration of ineffective or inefficient trial‐and‐error strategies in practice for analyzing targets with small distinctions from non‐targets. c) Schematic illustration of dynamic addressing analysis in our work for accurate and efficient target analysis.

Accurate and efficient molecular recognition serves as the cornerstone in the fields of molecular detection and molecular diagnostics.^[^ ^1^ ^]^ Previous strategies for target analysis in complex systems based on molecular recognition rely on trial‐and‐error approaches in which non‐targets that bind to the recognizers, or targets that bind to the non‐recognizers can be reversibly released until the correct molecule recognition ^[^ ^2^ ^]^ (**Figure** **1**a). However, when the distinction between target and non‐target is minimal, for example, single nucleotide polymorphism (SNP) of oligonucleotides, there are two critical challenges that arise (Figure 1b). One is that the incorrect recognition may not be reversible, resulting in ineffective trial‐and‐error and inaccurate analysis results, such as an incorrect antibodies recognition ^[^ ^3^ ^]^; the other is that even after successful reversion, rebinding efficiency to the correct target is hindered by Brownian movement, low target concentrations, etc., resulting in inefficient trial‐and‐error and suboptimal analysis results, such as complementary base pairing of short oligonucleotides.^[^ ^4^ ^]^ These problems are the key factors that lead to low accuracy and efficiency of analysis.

To address this challenge, current feasible solutions focus on the screening and application of more specific recognizers. One example is in protein analysis, the phosphorylation substructure of a given protein can be targeted by combining two antibodies to increase specificity. One antibody recognizes the protein itself, while the other recognizes the phosphorylation site.^[^ ^5^ ^]^ For another example, in nucleic acid analysis, the SNP can be detected by introducing locked nucleic acid (LNA) probes to increase specificity. Due to the unique rigid bicyclic structure, LNA exhibits higher thermodynamic stability than natural DNA probes in base pairing, resulting in a stronger binding affinity to target sequences in an application for microRNA fluorescence in situ hybridization (FISH),^[^ ^6^ ^]^ etc. In addition, the CRISPR‐Cas systems, which comprise a specific combination of CRISPR‐RNA (crRNA) and Cas nucleases, have also been used for nucleic acid analysis in recent years. Previous studies based on the CRISPR‐Cas systems have increased the specificity in target sequence recognition by improving the design of crRNA or the performance of Cas nucleases, which have been used for SNP identification.^[^ ^7^ ^]^ However, the above strategies heavily rely on specific recognizers, which increases practical difficulty, reduces universality, and has no substantial breakthrough in the basic principle of trial‐and‐error approaches.

It should be noted that the process of trial‐and‐error in solution relies on Brownian movement and random collisions between the molecules. The probability of collisions is closely related to the concentration of molecules. Lower concentrations of molecules result in lower collision probabilities, leading to increased costs of trial‐and‐error.^[^ ^8^ ^]^ If the recognizer is designed as a micro‐interface in solution to ensure the “Correct recognition” of the target, then performing the trial‐and‐error locally at the micro‐interface can greatly reduce the effects of Brownian movement and low of molecule concentrations.^[^ ^9^ ^]^ For this concept, the DNA walker is a successful practical case. As a typical representative of dynamic DNA devices, a DNA walker can achieve directional or non‐directional motion along the orbital strands under entropy, enthalpy, or chemical drive, exhibiting unique dynamic properties.^[^ ^10^ ^]^ Unfortunately, current strategies have mainly been studied as concepts or in the application of signal amplification, rather than for use in increasing specificity. Therefore, it remains a valuable area for the exploration of molecular devices based on this concept as well as their potential for the enhancement of specificity.

Inspired by the above, here we propose a concept of dynamic addressing analysis to achieve process specificity, a source of specificity depends not only on specific recognizers but on the process of recognition (Figure 1c). In this concept, we divide the target into the conserved (identical or similar to the non‐target) and the specific (distinct from the non‐target) region. Accordingly, the recognizer is divided into the conserved recognized region and the specific recognized region. The recognizer first binds to the conserved region of the target (i.e., the large central circle), while dynamically searching for the specific region of the target until a suitable match is achieved. This innovative concept has the potential to solve the two problems mentioned above. First, since the target and non‐target share conserved regions, the similarity between target and non‐target will no longer lead to incorrect recognition. Instead, specific differences in the specific region of the target can be determined by addressing, avoiding ineffective trial‐and‐error. In addition, there is no need for reversible release after incorrect recognition, eliminating the need for dissociation into a free state and thus avoiding inefficient trial‐and‐error.

To realize the proposed concept, we plan to develop a dynamic addressing molecular device for accurate analysis of the target. Molecular devices are devices constructed from molecular materials at the molecular scale or larger.^[^ ^11^ ^]^ Due to the powerful programmability, predictability, and structural stability, DNA has been widely used in the construction of molecular devices, especially dynamic molecular devices.^[^ ^12^ ^]^ Under the condition of reasonable design of functional regions, dynamic DNA devices can also exhibit excellent intelligence.^[^ ^13^ ^]^ The highly specific DNA base complementarity also provides thermodynamic tuning properties that are extremely conducive to the dynamic addressing of DNA devices.^[^ ^14^ ^]^ In this work, we have developed a dynamic addressing DNA device, named Dynamic Addressing Molecular Robot (DAMR), which includes a DNA framework scaffold, a conserved recognized region, and multiple specific recognized regions. The DAMR is designed to accurately address targets with low resolution in complex systems, such as SNP of microRNA (miR‐SNP). The DAMR can be further applied to analyze the SNP of miRNA in the cells and serum samples, enriching the current toolbox of SNP analysis, which is relatively scarce.^[^ ^15^ ^]^ The DAMR not only achieves the integration of structure and function, further expanding the various application of molecular devices, but also overcomes the deficiencies of the previous trial‐and‐error, providing a broad prospect for accurate and efficient analysis.

## 2 Results

### 2.1 Dynamic Addressing Analysis of miR‐SNPs Using DAMR

**Figure 2:**
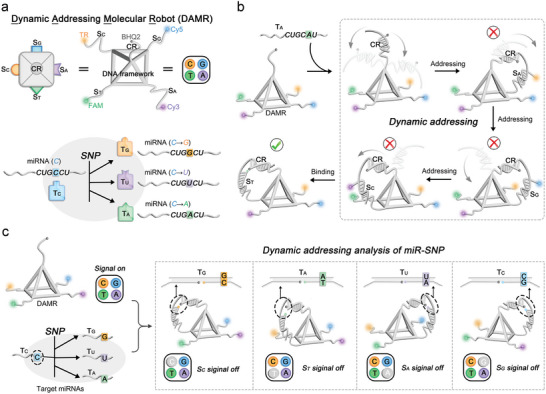
Schematic illustration of dynamic addressing analysis of miR‐SNPs using DAMR. a) Schematic illustration of the dynamic addressing molecular robot (DAMR) and four types of miR‐SNPs as the targets. b) Schematic illustration of the dynamic addressing process of DAMR for T_A_ analysis. c) Schematic illustration of the dynamic addressing analysis of multiple different miR‐SNPs in complex systems using DAMR.

We have designed a pyramid‐shaped DAMR to identify the different miR‐SNPs based on a trial‐and‐error strategy, in which all vertices carry a single‐stranded DNA through complementary pairing. As a dynamic addressing device, the apex at the top of the DAMR contains a quencher‐modified DNA as a conserved recognized region (CR) for binding to the conservative sequence of miRNAs. Meanwhile, four vertices at the bottom of the DAMR contain four fluorophore‐modified specific recognized regions (S) that are named S_A_ (Cy3‐modified), S_C_ (TR‐modified), S_G_ (Cy5‐modified), and S_T_ (FAM‐modified), respectively, which as the dynamic addressing arms of the robot to bind to the specific regions of miRNAs. We used a model containing the C base as a correct miRNA target model (T_C_), and targets with C mutated to A (T_A_), G (T_G_), or U (T_U_) as three types of interfering miR‐SNP targets (**Figure** **2**a). Each S of DAMR with a sequence that differs by only one base and can be used for identification with different types of miR‐SNPs. The dynamic addressing principle of DAMR for miR‐SNPs analysis is shown in Figure 2b. In the absence of the four types of miR‐SNPs, all four fluorophores carried by DAMR can be detected, resulting in DAMR being in a “signal on” state. When the target, such as T_A_, is present, the CR of DAMR binds to the conserved region of the T_A_ by complementary base pairs. Dynamic addressing then occurs, with the specific region of T_A_ wiggling until it finds and binds to the correct S_T_ by trial and error. The FAM modified on the S_T_ is quenched due to the proximity to the quencher, while the other fluorophores remain unchanged, causing DAMR to switch to an “S_T_ signal off” state. By monitoring the changes in fluorescence intensity of DAMR, we can accurately and efficiently determine the positive or negative presence of corresponding miR‐SNPs. Under this principle, DAMR can be used to simultaneously identify multiple different miR‐SNPs in complex systems by dynamic addressing analysis (Figure 2c).

### 2.2 Feasibility and Performance Study of Dynamic Addressing Analysis for miR‐SNP Analysis

DAMR was synthesized by self‐assembly of 9 single‐stranded DNA chains layer‐by‐layer. The scaffold of DAMR is a rigid DNA pyramid with a side length of 30 bp, while the CR at the top consists of a single‐stranded DNA. The four S at the bottom are all composed of single‐stranded DNA. The stepwise assembly of DAMR was verified by agarose gel electrophoresis (Figure S1, Supporting Information). The characterization results by atomic force microscopy (AFM) and dynamic light scattering (DLS) showed that DAMR had a size of ≈26.5 nm and a hydrodynamic diameter of ≈50.7 nm (Figures S2 and S3, Supporting Information). The 3D pyramidal shape of DAMR was observed by cryo‐electron microscopy (cryo‐EM) analysis (Figure S4, Supporting Information). These results indicated the successful synthesis of DAMR.

**Figure 3:**
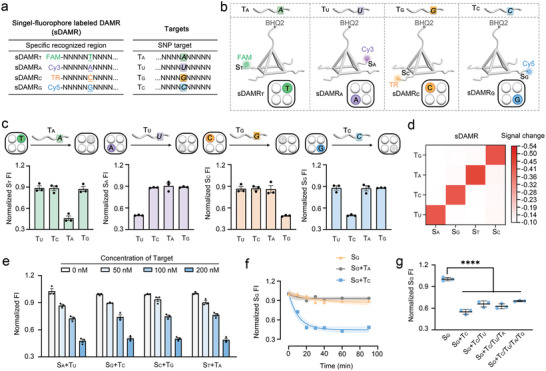
miR‐SNP analysis using single‐fluorophore labeled DAMR. a) Sequence information table of single‐fluorophore labeled DAMR (sDAMR) and the corresponding targets. b) Schematic illustration of four sDAMR and the corresponding targets. c) Normalized fluorescence intensity of four sDAMR after the addition of the different miR‐SNP targets, separately. Data are the means ± SD, *n* = 3. d) Signal changes of four sDAMR after the addition of the different miR‐SNP targets calculated from the data in c). e) Normalized fluorescence intensity of four sDAMR in response to different concentrations of the corresponding target. Data are the means ± SD, *n* = 3. f) Time‐dependent signal changes of Cy5‐labeled S_G_ before and after the addition of the target T_C_ or non‐target T_A_. Shaded bands are 95% confidence interval, *n* = 3. g) Normalized fluorescence intensity of S_G_ after the addition of the different targets to investigate anti‐interference capabilities. Data are the means ± SD, *n* = 3. *p* values were calculated by the one‐way ANOVA, ^****^ *p* < 0.0001.

To initially validate our reasonable speculations on the function of DAMR, several mimic targets were introduced to explore the dynamic addressing capabilities of DAMR. As shown in **Figure** **3**a,b, we first synthesized four single‐fluorophore labeled DAMR (sDAMR) for the analysis of the miR‐SNPs in which only one of the bases was artificially altered for the four targets, while the remaining bases were identical. To simultaneously analyze multiple targets in one pot, four fluorophores with distinctive excitation (Ex) and emission (Em) wavelengths (FAM: Ex/Em = 494/518 nm, Cy3: Ex/Em = 552/570 nm, Cy5: Ex/Em = 643/667 nm, and TR: Ex/Em = 595/615 nm; Figure S5, Supporting Information) were used for signal output. BHQ2 is a commonly used fluorescence quencher capable of quenching fluorescence signals emitted in the range of 520–650 nm.^[^ ^16^ ^]^ The results in Figure S6 (Supporting Information) showed that BHQ2 has an excellent quenching effect on Cy5, TR, Cy3, and FAM, which provides feasibility for subsequent analysis of targets by monitoring changes in fluorescence intensity of DAMR.

We then investigated the addressability of DAMR toward specific targets. First, two DNA strands, one containing CR and the other containing S region, were mixed with the target in order to investigate whether CR and S were able to bind to the corresponding target by complementary base pairing. Results of the PAGE analysis showed that the new bands can be observed in the presence of the corresponding target, which indicated the complementary binding of CR and S with the target (Figure S7, Supporting Information). Similarly, the results of the fluorescence intensity analysis showed a significant signal change due to the proximity of the fluorophore at the S terminal to the quencher at the CR terminal in the presence of the corresponding target (Figure S8, Supporting Information). Then, we optimized the temperature for dynamic addressing analysis. The signal variation between the presence and absence of the target is maximum at a reaction temperature of 25 °C, indicating that 25 °C is the optimal analysis temperature for efficient discrimination between target and non‐target (Figure S9, Supporting Information). Furthermore, a significant signal variation at the physiological temperature of 37 °C provides support for the reliability of DAMR in the analysis of cell samples. In addition, we investigated the effect of the length of the CR and S on the signal variation in response to the target. Results showed that the DAMR exhibited the maximum signal change in the presence of the target when the length of the CR is 11 nt and the length of S is also 11 nt, indicating optimal recognition of the target (Figure S10, Supporting Information).

We then introduced four miR‐SNP targets into the corresponding pre‐synthesized sDAMR. As shown in Figure 3c, S_T_ (modified with FAM) only responds to T_A_, while it does not exhibit strong interactions with T_C_, T_U_, or T_G_, with only slight fluctuations observed. We speculate that these fluctuations occur because during the addressing process, T_C_, T_U_, and T_G_ can also partially complement the specific region of the sDAMR for trial‐and‐error. Once complemented, the quencher has a certain probability of being pulled to the bottom, leading to varying degrees of quenching for even the non‐corresponding fluorophore. Similar situations are observed for the single‐target recognition of S_A_, S_C_, and S_G_. And we defined signal changes as:

(1) $$\mathrm{Signal}\;\mathrm{change}\;=\frac{\mathrm{FI}\;\left(\mathrm{after}\right)-\mathrm{FI}\;\left(\mathrm{before}\right)}{\mathrm{FI}\;\left(\mathrm{before}\right)}$$

where the FI (before) is the measured fluorescence intensity of DAMR before adding targets, FI (after) is the measured fluorescence intensity after adding targets. As shown in Figure 3d, the four sDAMR exhibit significant signal changes when the corresponding target is present, indicating the high specificity of DAMR in dynamic addressing analysis. These results demonstrate that The DAMR modified with a single fluorophore exhibits excellent addressability to a single target.

We then investigated the performance of DAMR for miR‐SNP analysis in terms of the different target concentrations, kinetics, and anti‐interference capabilities. As shown in Figure 3e, when the DAMR concentration is 200 nM, the fluorescence intensity decreases continuously as the target concentration increases from 0 to 200 nM. However, when the target concentration continues to increase far beyond the device concentration, the fluorescence intensity exhibits a recovery trend (Figure S11, Supporting Information). Based on this observation, we speculate that an excess of targets may hinder the addressing behavior of DAMR. These targets might compete for the top and bottom of the DAMR separately, rather than bringing the top and bottom closer together. In the kinetic study, we discovered that the best‐addressing efficiency is achieved when DAMR and the target are incubated for 20 min, and as the incubation time is extended, the fluorescence intensity remains at a plateau (Figure 3f). In addition, the control group without miR‐SNP targets was used to correct for potential signal variations in DAMR, thereby increasing to the accuracy of the analysis. In terms of anti‐interference, DAMR also performs well. Using S_G_ as the research platform for analyzing various targets, the results in Figure 3g demonstrate that as long as the target T_C_ is present, S_G_ can sort it out and output the correct signal. The presence of other non‐specific targets (such as T_U_ T_A_, or T_G_) will not significantly affect the change of the output signal. These results support our speculation about the performance of DAMR, which possesses superior dynamic properties and anti‐interference capabilities. Based on the “trial‐and‐error” operating principle, it returns to the correct track after recognizing interfering targets.

### 2.3 Multiple miR‐SNPs Analysis in Complex Systems Using DAMR

**Figure 4:**
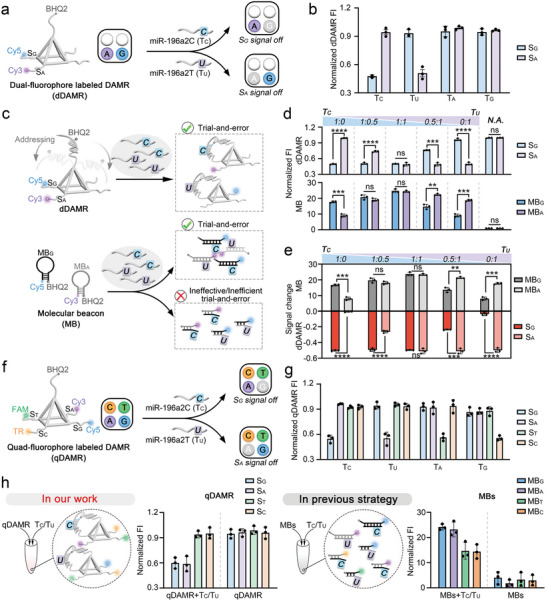
Multiple miR‐SNPs analysis using DAMR and comparison with previous trial‐and‐error approach. a) Schematic illustration of dual‐fluorophore labeled DAMR (dDAMR) for identifying the miR‐SNP targets. b) Normalized fluorescence intensity of S_G_ and S_A_ in dDAMR after the addition of the different miR‐SNP targets, separately. c) Schematic illustration of the comparison between dynamic addressing analysis using DAMR and the previous MB trial‐and‐error approach in target identification. d) Normalized fluorescence intensity of dDAMR or MBs in response to different ratios of miR‐SNP targets. e) Signal changes of dDAMR or MBs in response to different ratios of targets calculated from the data in d). f) Schematic illustration of quad‐fluorophore labeled DAMR for identifying the miR‐SNP targets. g) Normalized fluorescence intensity of four specific recognized regions (S) in DAMR after the addition of the different miR‐SNP targets, separately. h) Normalized fluorescence intensity of DAMR or MBs after the addition of the mixed miR‐SNP target system. Data are the means ± SD, *n* = 3. *p* values were calculated by the 2‐tailed student's *t*‐test, ^**^ *p* < 0.01, ^***^ *p* < 0.001, ^****^ *p* < 0.0001, ns means no significance.

Next, we evaluated the ability a of DAMR to identify SNP targets present in complex samples. Previous studies have shown that the SNP variation rs11614913 within the miR‐196a2 gene, known as miR‐196a2 T>C SNP, results in a mutation from C to U at the sixth base of the mature miRNA sequence. This mutation has been shown to affect the expression and regulatory functions of miRNA, thereby promoting the development of colorectal cancer, acute lymphoblastic leukemia, and non‐small cell lung cancer.^[^ ^17^ ^]^ To distinguish the miR‐SNPs, miR‐196a2 T (T_U_) and miR‐196a2 C (T_C_), we synthesized a dual‐fluorophore (Cy3 and Cy5 at the S_A_ and S_G_ regions, respectively) labeled DAMR (dDAMR) (**Figure** **4**a). As shown in Figure 4b, the signal intensity of Cy5‐labeled S_G_ in dDAMR was significantly reduced in the presence of miR‐196a2 T (T_U_). Similarly, the intensity of Cy3‐labeled S_A_ was also significantly reduced in the presence of miR‐196a2 C (T_C_). In contrast, no significant changes in Cy3 and Cy5 were observed when non‐targets T_A_ and T_G_ were added to the dDAMR. These results demonstrate the excellent selectivity of DAMR in identifying multiple targets, even in the presence of complex mixtures.

We then focus on comparing dynamic addressing analysis using DAMR with previous trial‐and‐error approaches. As an example, we performed miR‐SNPs analysis using classical molecular beacons (MBs), which are based on the principle of molecular recognition, and determined the presence of miR‐SNPs by detecting the fluorescent signals emitted by the beacons.^[^ ^18^ ^]^ We performed the analysis of miR‐196a2 T and miR‐196a2 C using DAMR and fluorescence‐labeled MB mixed systems, respectively (Figure 4c). We selected Cy3‐labeled MB_A_ and Cy5‐labeled MB_G_ to detect T_U_ and T_C_ as miR‐SNP targets, respectively, while T_A_ and T_G_ served as non‐target controls. As shown in Figure S12 (Supporting Information), the significant fluorescence change was observed with MB_A_ when T_U_ was added. It is noted that fluorescence changes were also observed to varying degrees when T_C_, T_A_, or T_G_ was added to MB_A_. Similarly, MB_G_ showed significant fluorescence changes when T_C_ was present, but also showed responses to the T_U_, as well as the non‐target controls T_A_ and T_G_. It is obviously opposite to the results obtained from the DAMR analysis. Our previous results have already demonstrated the accuracy of DAMR in responding to targets, as the signals generated by DAMR do not change significantly in the presence of non‐targets (Figures 3c and 4b).

We further expanded our investigation by introducing different ratios of miR‐SNPs in dDAMR and MBs, respectively. As shown in Figure 4d,e, the changes in fluorescence intensity of dDAMR were clearly correlated with the ratio of miR‐SNPs. When the miR‐SNP target ratio (either T_C_: T_U_ or T_U_: T_C_) was 1:0, significant differences in the signal changes of S_G_ or S_A_ were observed. As the miR‐SNP target ratio approached 1:1, the signal changes of S_G_ and S_A_ were approximately the same. These results indicated that monitoring the signal changes of DAMR accurately reflected the presence of corresponding targets in the system. However, in the MBs, non‐specific signals inconsistent with the corresponding target ratios were consistently found, indicating that the selectively analytical capability of MBs in complex multi‐target systems is significantly lower than that of DAMR. We speculated that the disparity in performance between MB and DAMR in miR‐SNP analysis was due to the single, non‐dynamic structure of MB, which prevented it from returning to the stem‐loop state after the misidentification (Figure 4c). In contrast, DAMR, which is characterized by conserved and specific recognized regions, exhibits a dynamic addressing capability, allowing direct and accurate target identification in a single step. In addition, the probability of molecular collisions of MB and DAMR in solution is theoretically consistent. The DAMR could be considered as a micro‐interface containing multiple recognizers in solution, which allows for the specific recognition and accurate sorting of targets. However, conventional methods, such as molecular beacons, have a lower specificity and accuracy in target recognition due to the ineffective and inefficient trial‐and‐error process.

We further performed the analysis of the miR‐SNPs using quad‐fluorophore (Cy3, Cy5, TR, and FAM) labeled DAMR (Figure 4f). We first demonstrated that DAMR labeled with quad‐fluorophore still retains excellent addressability by analyzing single targets (Figure 4g). Afterward, as shown in Figure 4h, in the presence of both targets miR‐196a2 T (T_U_) and miR‐196a2 C (T_C_), the signal intensities of Cy3‐labeled S_A_ and Cy5‐labeled S_G_ in DAMR were significantly changed, while that of FAM‐labeled S_T_ and TR‐labeled S_C_ were almost unaffected. When the same operation was performed using the four fluorescence‐labeled MBs, all the signal intensities of MBs changed in the presence of both T_U_ and T_C_, although MB_A_ and MB_G_ showed the most significant changes. The dynamic addressing process of DAMR was further studied by adding the T_U_ and T_C_ in sequence. As shown in Figure S13 (Supporting Information), after the addition of the T_U_ and T_C_, the fluorescence intensities of S_A_ and S_G_ were gradually changed and reached equilibrium within 20 min, while those of S_A_ and S_G_ remained unchanged. These results indicate that DAMR, equipped with four dynamic addressable recognizers, has the remarkable ability to recognize corresponding targets simultaneously in one step via the dynamic addressing process. The characteristic of the dynamic addressable recognition has provided the efficiency and convenience of DAMR, providing an effective and efficient approach to trial‐and‐error in complex systems.

### 2.4 Analysis of miR‐SNPs in Cells and Serum Samples Using DAMR

Currently, there are several methods for detecting miR‐SNPs at the genomic level, but the detection of miR‐SNPs at the transcriptomic level has not received sufficient attention, which hinders disease screening. Considering the excellent performance of DAMR in analyzing miR‐SNPs, we expanded our research to include the detection of miR‐SNPs in cells and blood samples to explore the potential applications of DAMR.

**Figure 5:**
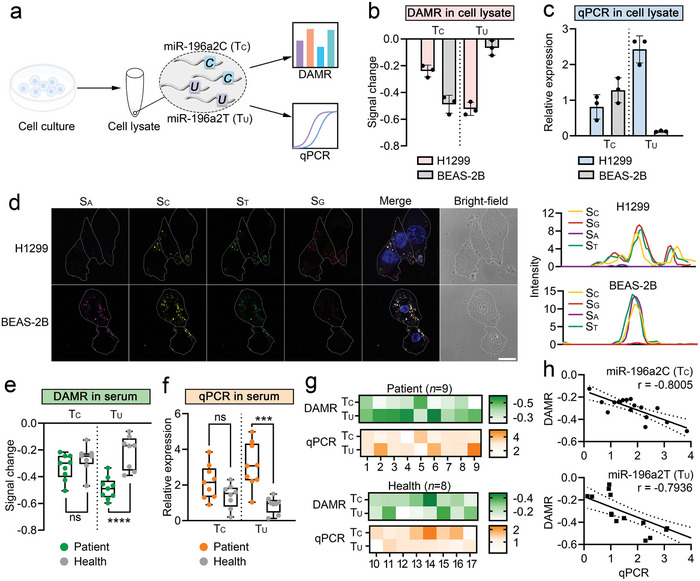
miR‐SNP analysis in cells and serum samples using DAMR. a) Schematic illustration of DAMR and qPCR for miR‐SNPs analysis at the cellular level. b) Signal changes of DAMR for analysis of miR‐SNP targets in H1299 and BEAS‐2B cell lysates. *n* = 3. c) Relative expression of miR‐SNP targets in H1299 and BEAS‐2B cell lysates using qPCR. *n* = 3. d) Confocal fluorescence imaging of H1299 and BEAS‐2B cell with the transfection of DAMR. Scale bar = 20 µm. e) Signal changes of DAMR for analysis of miR‐SNP targets in serum samples from NSCLC patients (*n* = 9) and healthy individuals (*n* = 8). f) Relative expression of miR‐SNP targets in serum samples from NSCLC patients (*n* = 9) and healthy individuals (*n* = 8). g) Heat maps of signal changes measured by DAMR and relative expression measured by qRT‐PCR. h) Correlation evaluation of DAMR and qRT‐PCR, respectively. Pearson's *r* for miR‐196a2 C (T_C_) = −0.8005, P = 0.0001, and *r* for miR‐196a2 T (T_U_) = −0.7936, P = 0.0001. Data are the means ± SD, *p* values were calculated by the 2‐tailed student's *t*‐test, ^**^ *p* < 0.01, ^***^ *p* < 0.001, ^****^ *p* < 0.0001, ns means no significance.

We initiated the analysis of miR‐SNPs using DAMR at the cellular level (**Figure** **5**a). Previous research indicates that the miR‐196a2 T>C SNP is associated with individual survival in non‐small cell lung cancer (NSCLC) patients.^[^ ^17b^ ^]^ Here, H1299 cells (human NSCLC cells) and BEAS‐2B cells (human normal lung epithelial cells) were first cultured to the exponential growth phase and lysed. We extracted the total cellular miRNA from the cell lysates. We then assessed the miR‐196a2 T (T_U_) and miR‐196a2 C (T_C_) in both cell types by introducing DAMR and analyzing the signal changes of S_A_ and S_G_. As shown in Figure 5b, the S_A_ signal indicating T_U_ content was significantly changed in H1299 cells, while minimal change was observed in BEAS‐2B cells. On the other hand, the S_G_ signal indicating T_C_ content was changed in both cell types, with a larger change exhibited in BEAS‐2B cells compared to H1299 cells. Next, quantitative real‐time polymerase chain reaction (qRT‐PCR) of T_U_ and T_C_ in both cell types provided similar results, confirming the accuracy of DAMR for analysis of miR‐SNPs at the cellular level (Figure 5c). We further transfected DAMR into BEAS‐2B and H1299 cells through transfection reagents, such as lipofectamine, for in situ imaging analysis of T_U_ and T_C_. Consistently, the corresponding changes in fluorescence intensity of S_A_ or S_G_ were observed in the cytoplasm of both cell types (Figure 5d). Furthermore, the distribution of T_U_ and T_C_ in both cell types was investigated by fluorescence in situ hybridization (FISH) (Figure S14, Supporting Information). It is concerned that the localization of DAMR might be influenced by the diffusion of targets, whereas the dynamic addressing process is an intramolecular reaction that would not be influenced. These results indicated a higher expression level of miR‐196a2 T>C SNP in H1299 cells compared to the normal BEAS‐2B cells.

We further investigated the analysis of miR‐SNPs using DAMR in serum samples to evaluate the potential applications in clinical assays. We evaluated the stability of DAMR in untreated serum before the analysis. Result of agarose gel electrophoresis showed that the structure of DAMR did not change significantly within 6 h in serum, indicating that DAMR could be used for miR‐SNPs analysis in serum for at least 6 h (Figure S15, Supporting Information). It is consistent with previous studies that DNA frameworks can maintain structural stability in serum for several hours.^[^ ^19^ ^]^ We then tested the extracted small RNA in serum samples from NSCLC patients (*n* = 9) and healthy individuals (*n* = 8) using DAMR and qRT‐PCR assays. As shown in Figure 5e, DAMR analysis revealed that the S_A_ signal changes were greater in patient samples compared to those of healthy control samples (*p* < 0.01), whereas the S_G_ signal changes were similar to those of the controls. These results indicated that the expression level of miR‐196a2 T (T_U_) in NSCLC patients was significantly higher than in healthy individuals, and also suggested that DAMR has the ability to identify more miR‐SNP targets in clinical samples. Similar conclusions were also obtained using qRT‐PCR for measuring the expression level of T_U_ and T_C_ in clinical samples (Figure 5f). We further compared the results obtained by the DAMR and qRT‐PCR (Figure 5g,h). The results demonstrated a moderate correlation, with a Pearson's *r* of −0.8005 for miR‐196a2 C (T_C_) and −0.7936 for miR‐196a2 T (T_U_). It may be attributed to the reduction in accuracy during the reverse transcription and amplification steps in the qRT‐PCR process, which results in a moderate correlation between the results obtained by the DAMR and qRT‐PCR.^[^ ^20^ ^]^ Finally, we investigated the analysis of miR‐SNPs using DAMR in the untreated serum samples (Figure S16, Supporting Information). However, the signal changes observed in the analysis of untreated serum samples were not statistically significant. It may be attributed to the low RNA concentration in the untreated serum, which resulted in DAMR not having sufficient sensitivity to analyze. It is worth mentioning that the whole procedure of DAMR analysis requires only 20 min of incubation with the samples, followed by statistics of changes in fluorescence intensity, while qRT‐PCR takes ≈2 h. Moreover, DAMR has lower synthetic running costs than qRT‐PCR, making it an attractive candidate for clinical diagnostic applications that could help with miR‐SNP‐related disease screening.

## 3 Conclusion

Molecular recognition is crucial for molecular detection and diagnostics. However, accurate and efficient recognition of targets in complex systems remains a challenge. Here, we propose a concept of dynamic addressing analysis by designing a Dynamic Addressing Molecular Robot (DAMR) to accurately and efficiently distinguish multiple targets that have minimal distinctions in complex systems. DAMR overcomes the current challenge in recognition by achieving dynamically specific recognition. In the presence of the target, DAMR can first capture the target through its conserved recognition region (CR) and then activate dynamic addressing to allow the target to search for and ultimately bind to the appropriate specific recognition region (S), resulting in the corresponding signal changes. The DAMR has demonstrated higher accuracy and efficiency compared to the traditional trial‐and‐error approach for miR‐SNPs analysis. DAMR can be applied to analyze the SNPs of miR‐196a2 in cells and serum samples, the results obtained are consistent with those obtained using the current qRT‐PCR, indicating that the dynamic addressing analysis we have constructed has good detection performance.

The development of DAMR not only provides a novel tool for the analysis of miR‐SNPs but also provides new insights into the design of DNA framework. Future research could focus on improving the specificity of DAMR toward certain types of miR‐SNP targets by optimizing the design of the CR and S regions. In addition, the exploration of novel output signal types, such as the introduction of “signal‐on” probes designed by highly programmable DNA nanostructures or DNA encoding, could also enhance the versatility and applicability of DAMR for analysis. In comparison to the “signal‐off” strategy, the “signal‐on” strategy showed significant advantages in integrating signal amplification strategies, such as electrochemical or nanoparticle‐based sensing platforms, which could further improve the sensitivity of the analysis. Therefore, future research could explore the integration of “signal‐on” probes and DAMR to advance the development and application of dynamic addressing analysis in the diagnostic field. Continued advances in nanotechnology and nucleic acid analysis have the potential to integrate DAMR into miniaturized, portable diagnostic devices for point‐of‐care testing, which is also an exciting direction for future research.

## Conflict of Interest

The authors declare no conflict of interest.

## Supporting information

Supporting Information

## Data Availability

The data that support the findings of this study are available in the supplementary material of this article.
