# Supplementary material for: Dynamic Addressing Molecular Robot (DAMR): An Effective and Efficient Trial‐and‐Error Approach for the Analysis of Single Nucleotide Polymorphisms
Source: Adv Sci (Weinh). 2024 Jun 17;11(31):2402140. doi: 10.1002/advs.202402140 (PMC11336946; doi:10.1002/advs.202402140)
Supplement: Supplementary file 1 — Supporting Information [file ADVS-11-2402140-s001.docx]

Supporting Information

Dynamic Addressing Molecular Robot (DAMR): An Effective and Efficient Trial-and-Error Approach for the Analysis of Single Nucleotide Polymorphisms

Tianshu Chen, Guifang Chen, Siyu Cao, Xiaochen Tang, Wenxing Li, Chenbin Liu，Hongquan Gou, Pei Sun, Yichun Mao, Qiuhui Pan^*^, Penghui Zhang^*^, Xiaoli Zhu^*^

Table of Contents

**Experimental Procedures3**

**Results7**

Figure S17

Figure S27

Figure S38

Figure S48

Figure S59

Figure S69

Figure S710

Figure S810

Figure S911

Figure S1011

Figure S1112

Figure S1212

Figure S1313

Figure S1413

Figure S1514

Figure S1614

Table S115

**Experimental Procedures**

**Chemicals and materials**

All DNA oligonucleotides were synthesized and purified by Hippo Biotechnology Co., Ltd. (Huzhou, China). The sequences are shown in Table S1 4% paraformaldehyde (PFA), 4',6-diamidino-2-phenylindole (DAPI), and PBS were purchased from Sangon Biotechnology Co., Ltd. (Shanghai, China). TE (Tris-EDTA), magnesium chloride (MgCl_2_), sodium hydroxide (NaOH), and hydrochloric acid (HCl) were purchased from Shanghai National Pharmaceutical Reagent Co., Ltd. (Shanghai, China). All solutions were prepared using ultrapure water (18.2 MΩ•cm^-1^) produced by a Milli-Q purification system (Millipore Corp, Milford, MA). Other chemicals were of analytical grade and were purchased from Sigma Aldrich (St. Louis, MO, USA). All fluorescence signals were acquired at 25 °C using a xenon lamp excitation source on a Hitachi F-7000 spectrophotometer (Hitachi, Japan), with excitation wavelengths of 552 nm for Cy3, 643 nm for Cy5, 595 nm for TR (Texas Red), and 494 nm for FAM, and emission wavelengths of 570 nm, 667 nm, 615 nm, and 518 nm, respectively.

**Preparation and characterization of DAMR**

DAMR, single-fluorophore labeled DAMR (sDAMR), and dual-fluorophore labeled DAMR (dDAMR) were assembled through DNA base pairing interactions. In brief, 9 ssDNA strands were used with the sequences shown in Table S1. X_4_ strand was modified with BHQ-2, while S_A_, S_C_, S_G_, and S_T_ strands were respectively modified with four different fluorescent groups, Cy3, TR, Cy5, FAM, and the other strands were unmodified. Initially, the 9 strands were mixed in TE buffer (containing 12.5 mM MgCl_2_, pH 7.4) at a final concentration of 500 nM. The mixture was heated to 95 °C and gradually cooled down (5 mins from 95 °C to 80 °C, 2 min/°C from 80 °C to 60 °C, and then 3 min/°C from 60 °C to 4 °C). The assembled DAMR was then stored at 4 °C for futher experiments.

The assembly of DAMR was characterized by agarose gel electrophoresis. A volume of 5 μL and 500 nM of samples was mixed with 1 μL 6× loading buffer, and was loaded onto a 3% agarose gel. The electrophoresis experiment was performed in 1×TAE at 120 V for 25 min. The images were captured using a Bio-Rad Gel Doc XR Gel Imaging System (Bio-Rad, USA). The morphologies of the DAMR were determined by Agilent 5500 atomic force microscope (Agilent Technologies, USA). The size distribution was analyzed with a ZETASIZER 3000HS instrument (Malvern Instruments Ltd., UK). For the cryo-EM analysis, 1 μM of DAMR was dropped on 200 mesh R1.2/1.3 copper grid. The sample was then blotted with a FEI Vitrobot Chamber (Thermo Fisher Scientific, USA). The grid was then flash frozen in liquid ethane and transferred into liquid nitrogen. The sample was observed using a FEI Talos F200C cryo-electron microscope (Thermo Fisher Scientific, USA) at a 200 kV accelerating voltage.

**Operation of DAMR for miR-SNP analysis**

Four miR-SNP targets, which were nearly identical except for a single nucleotide difference, were shown in Table S1. For the operation of sDAMR, 200 nM of four different targets were mixed with 200 nM of pre-synthesized sDAMR respectively at 25 °C for 20 min. The fluorescent signals of each sDAMR before and after operation were analyzed using a Hitachi F-7000 spectrophotometer.

For the operation of dDAMR, different ratios of two miR-SNP targets, T_U_ and T_C_, were mixed with 200 nM of dDAMR (containing a Cy3-labeled S_A_ and a Cy5-labeled S_G_) simultaneously at 25 °C for 20 min. The ratio of T_U_ and T_C_ includes 1:0 (200 nM of T_U_ and 0 nM of T_C_), 1:0.5 (200 nM of T_U_ and 100 nM of T_C_), 1:1 (200 nM of T_U_ and 200 nM of T_C_), 0.5:1 (100 nM of T_U_ and 200 nM of T_C_), and 0:1 (0 nM of T_U_ and 200 nM of T_C_). The fluorescent signals of S_A_ and S_G_ in dDAMR before and after operation were analyzed using a spectrophotometer.

For the operation of quad-fluorophore labeled DAMR, 200 nM of miR-SNP targets were mixed with 200 nM of DAMR at 25 °C for 20 min, followed by the fluorescence intensity measurement of Cy3, Cy5, TR and FAM in DAMR using a spectrophotometer.

**Operation of molecular beacon for miR-SNP analysis**

Four molecular beacons (MBs) were used for comparison with DAMR. 200 nM of miR-SNP targets were added into a mixture containing 200 nM of MB_T_, MB_G_, MB_C_, and MB_A_ at 25 °C for 1 h. For comparison with dDAMR, different ratios of T_U_ and T_C_, were added into a mixture containing 200 nM of MB_A_ and MB_G_ at 25 °C for 1 h. Then, the fluorescence intensity was measured using a spectrophotometer.

**Cell culture**

Human non-small cell lung cancer cells (H1299) and human normal lung epithelial cells (BEAS-2B) were obtained from the Institute of Biochemistry and Cell Biology (Chinese Academy of Science). H1299 cells were cultured in RPMI-1640 (Gibco, Invitrogen) supplemented with 10% FBS (Gibco, Invitrogen), 1% penicillin and streptomycin. BEAS-2B cells were cultured in DMEM medium (Gibco, Invitrogen) supplemented with 10% FBS, 1% penicillin and streptomycin. Cells were maintained in a humidified cell culture incubator at 37 ℃ with 5% CO_2_. Cells were collected using trypsin when they reached logarithmic growth phase for subsequent experiments.

**Analysis of miR-SNPs at the cellular level**

Total miRNAs were extracted from H1299 and BEAS-2B cells using the SanPrep column microRNA extraction kit (Sangon Biotechnology, China) according to the instructions. Then, 10 nM of quad-fluorophore labeled DAMR was added to 2 μg of miRNAs in cell lysates at 25 °C for 20 min. The fluorescent signals of S_A_ and S_G_ in DAMR were analyzed using a spectrophotometer to indicate the contents of the miR-196a2 T (T_U_) and miR-196a2 C (T_C_), respectively.

For the imaging of miR-SNPs *in situ*, H1299 and BEAS-2B cells were seeded in confocal dishes and incubated for 24 h. Then, 10 nM of quad-fluorophore labeled DAMR was delivered into cells using Hieff Trans liposome nucleic acid transfection reagent (Yeasen Biotechnology, China) at 37 ℃ for 4 h. After washing with T-PBS (PBS buffer containing 0.5% Tween-20) for three times, cells were fixed with 4 % PFA at room temperature for 10 min, and then stained with DAPI. After wishing, the observations of cells were performed under a LSM 710 confocal laser scanning microscope (Zeiss, Germany).

For the fluorescence in situ hybridization (FISH) analysis, H1299 and BEAS-2B cells were seeded in confocal dishes and incubated for 24 h. After washing, cells were fixed with 4 % PFA, and then dehydrated through a series of 70%, 85% and 99.5% ethanol for 3 min each. After washing, cells were treated with 100 nM FISH probes, 0.5 mg/mL yeast tRNA and salmon sperm DNA, and 40 U of RNase inhibitor in 2 × SSC buffer at 37 °C for 90 min. Then cells were stained with DAPI. The observations of cells were performed under a Leica TCS SP confocal laser scanning microscope (Leica, Germany).

**Analysis of miR-SNPs in serum samples**

Clinical serum samples were obtained from Shanghai Tenth People's Hospital (Shanghai, China). This study was approved by the ethics committee of Shanghai Tenth People's Hospital (23KT68). Written informed consent was obtained from every participant. Total miRNA was extracted from the serum samples using the SanPrep column microRNA extraction kit. 10 nM of quad-fluorophore labeled DAMR was added to 2 μg of miRNAs in serum samples at 25 °C for 20 min. The fluorescent signals of S_A_ and S_G_ in DAMR were analyzed using a spectrophotometer to indicate the contents of the miR-196a2 T (T_U_) and miR-196a2 C (T_C_), respectively.

**qRT-PCR assay**

Total miRNA was extracted from the cells or serum samples using the SanPrep column microRNA extraction kit according to the instructions. Then total RNA samples were reverse transcribed using HG TaqMan miRNA cDNA synthesis kit (HaiGene Biotech Co., Ltd, China) according to the instruction manual to obtain cDNA. U6 was used as endogenous control for miR-196a2. qRT–PCR analysis was performed with the cDNA using a HG TaqMan miRNA qPCR Kit on a CFX96 Real-Time System (Bio-Rad, USA). The relative expression of miR-196a2 T (T_U_) and miR-196a2 C (T_C_) were analyzed using the 2^-ΔΔCt^.

**Statistical analysis.** Fluorescence intensity was analyzed using Image J software. Results were presented as means ± SD, *n* = 3. *p* values were calculated by the 2-tailed student's *t*-test unless otherwise noted. **p* < 0.1, ***p* < 0.01, ****p* < 0.001, *****p* < 0.0001, ns means no significance.

**Results**


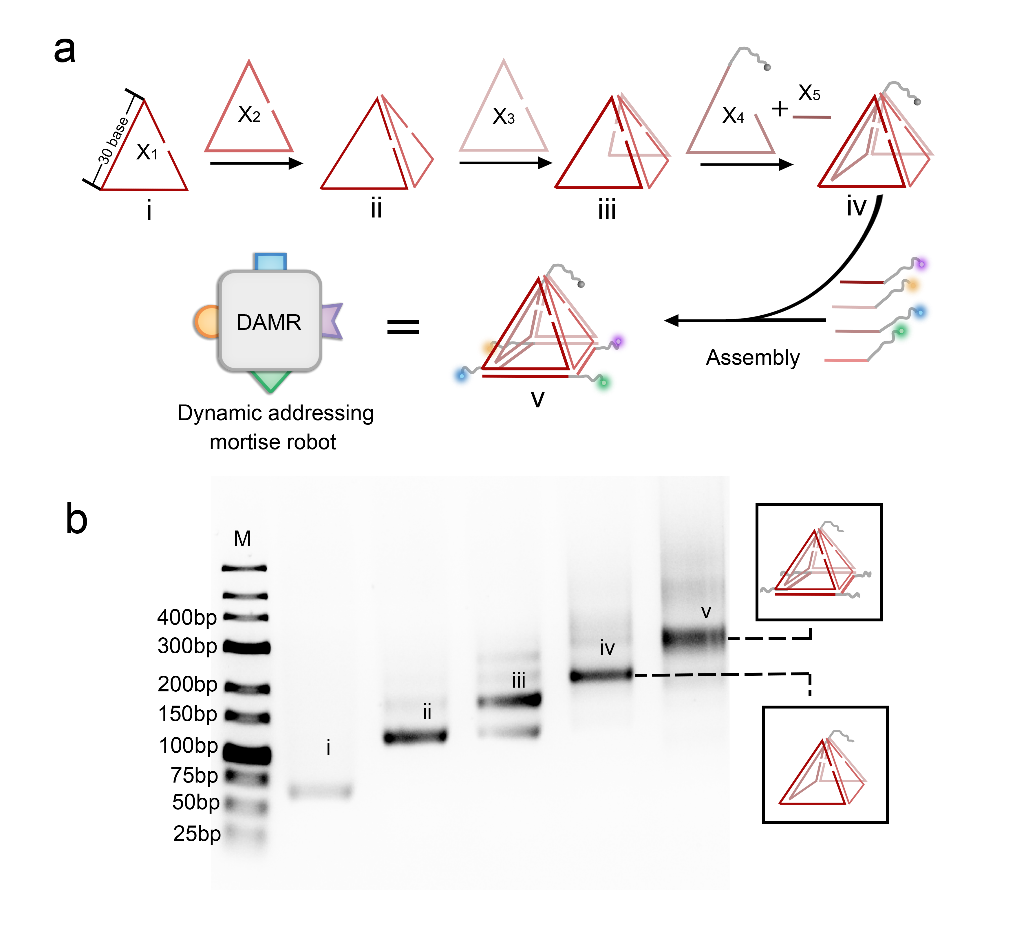


**Figure S1.** (a) Schematic illustration of the synthesis of DAMR by self-assembly. (b) Verification of the synthesis of DAMR by 3% agarose gel electrophoresis.


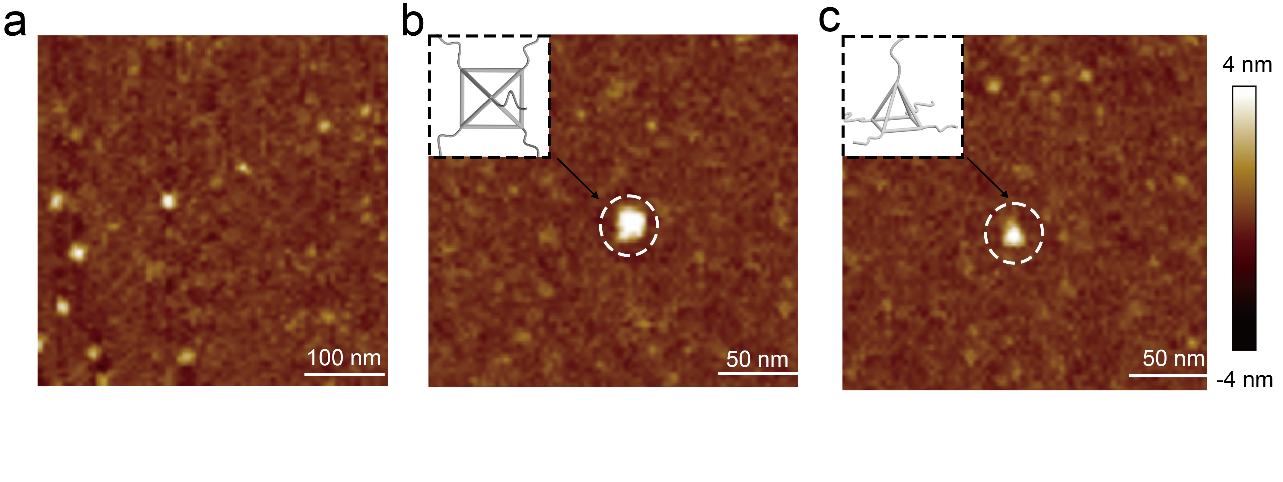


**Figure S2.** Atomic force microscopy (AFM) for characterization of DAMR on the overall (a), top (b), and side (c) view.


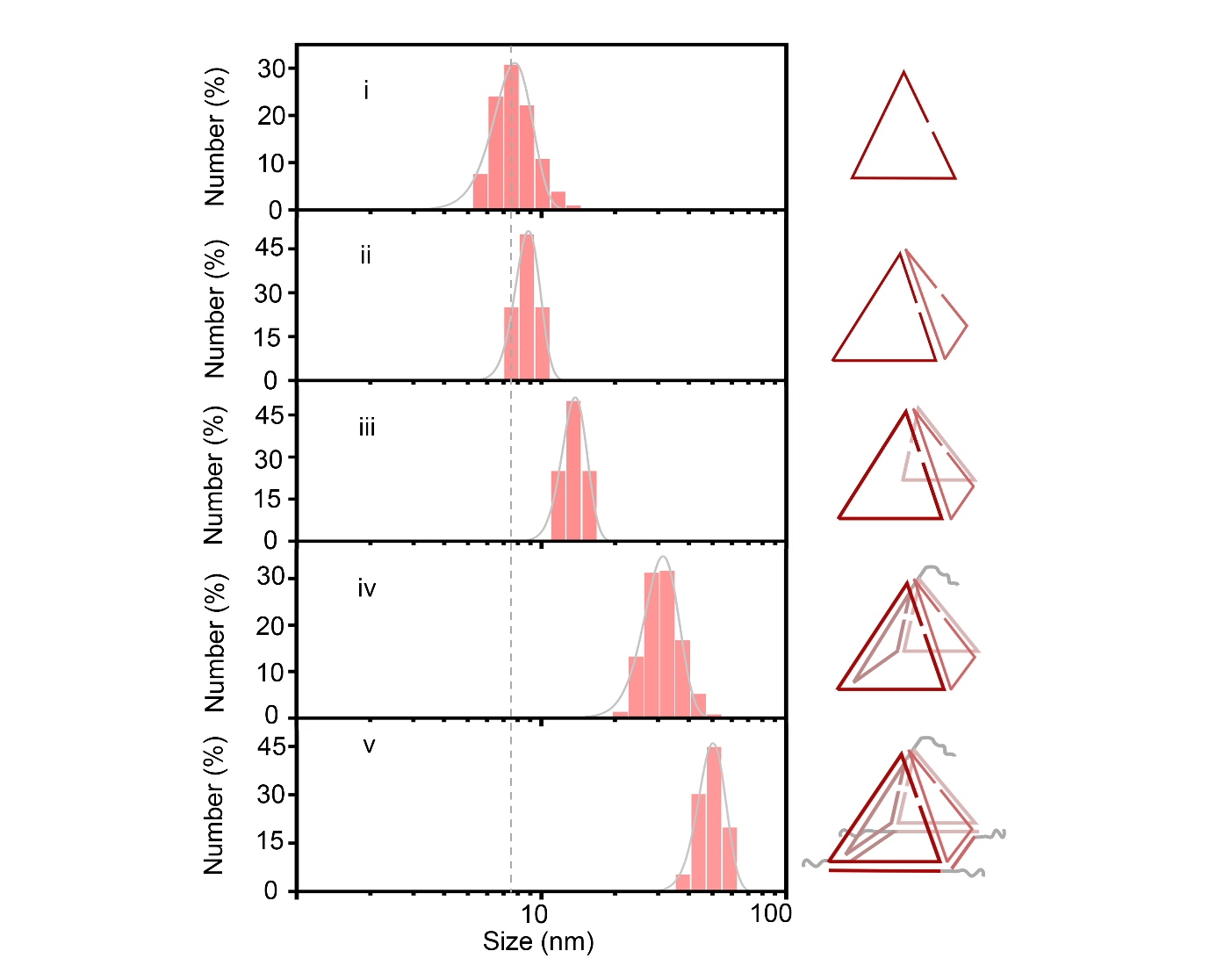


**Figure S3.** Dynamic light scattering (DLS) of the DAMR. The average hydrodynamic diameter of DAMR is 50.7 ± 12.9 nm.


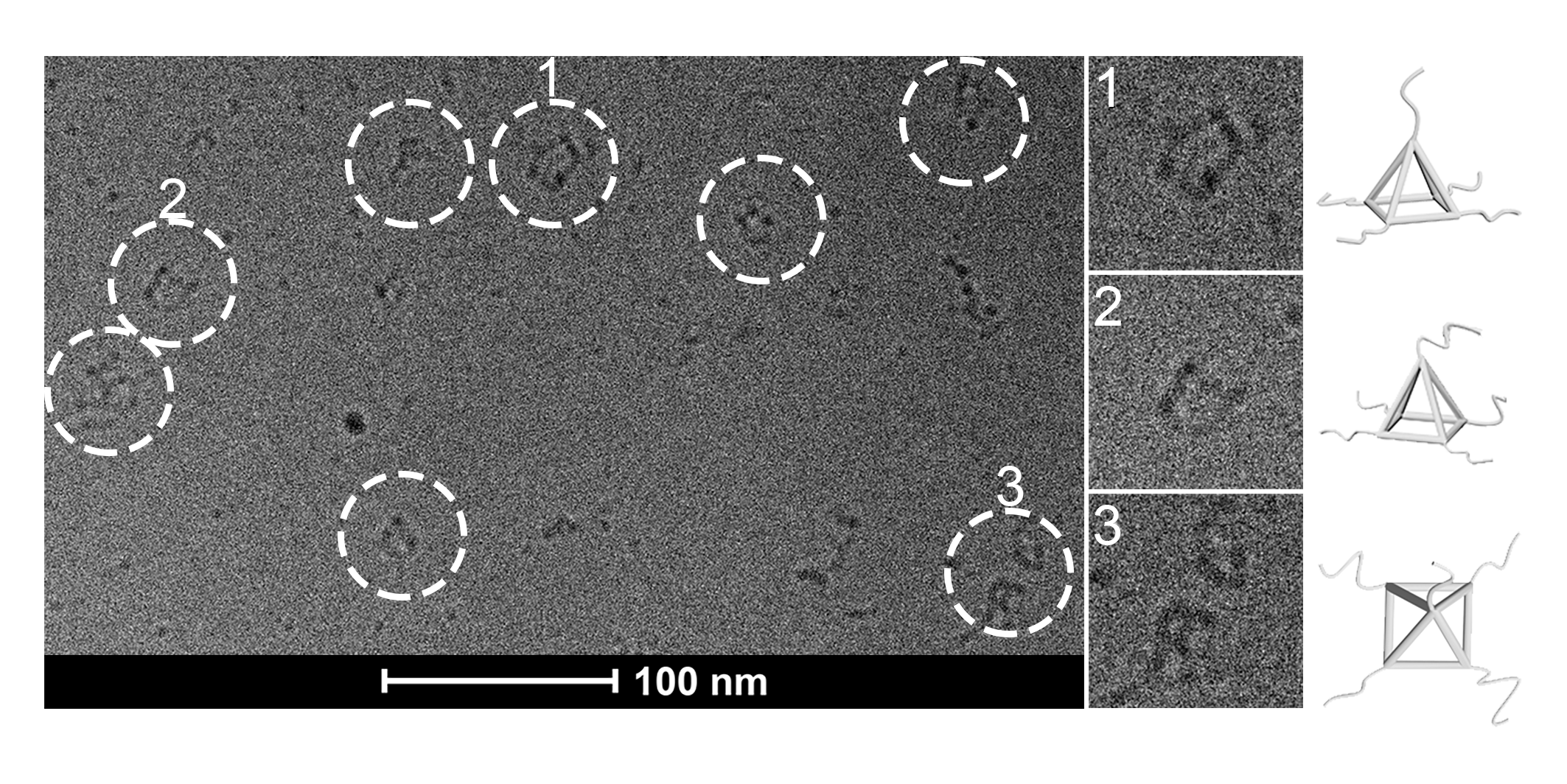


**Figure S4.** Cryo-electron microscopy (cryo-EM) analysis for characterization of the topological structure of DAMR.


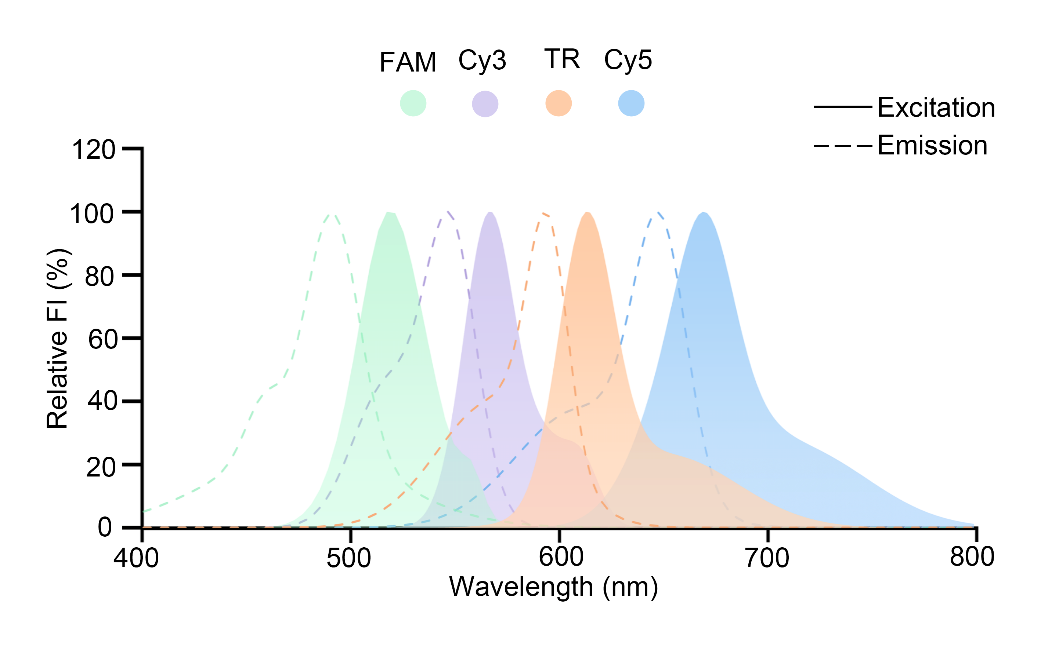


**Figure S5.** Excitation (Ex) and emission (Em) spectra of FAM, Cy3, TR and Cy5. FAM：Ex/Em = 494/518 nm; Cy3: Ex/Em = 552/570 nm; Cy5: Ex/Em = 643/667 nm; TR: Ex/Em= 595/615 nm.


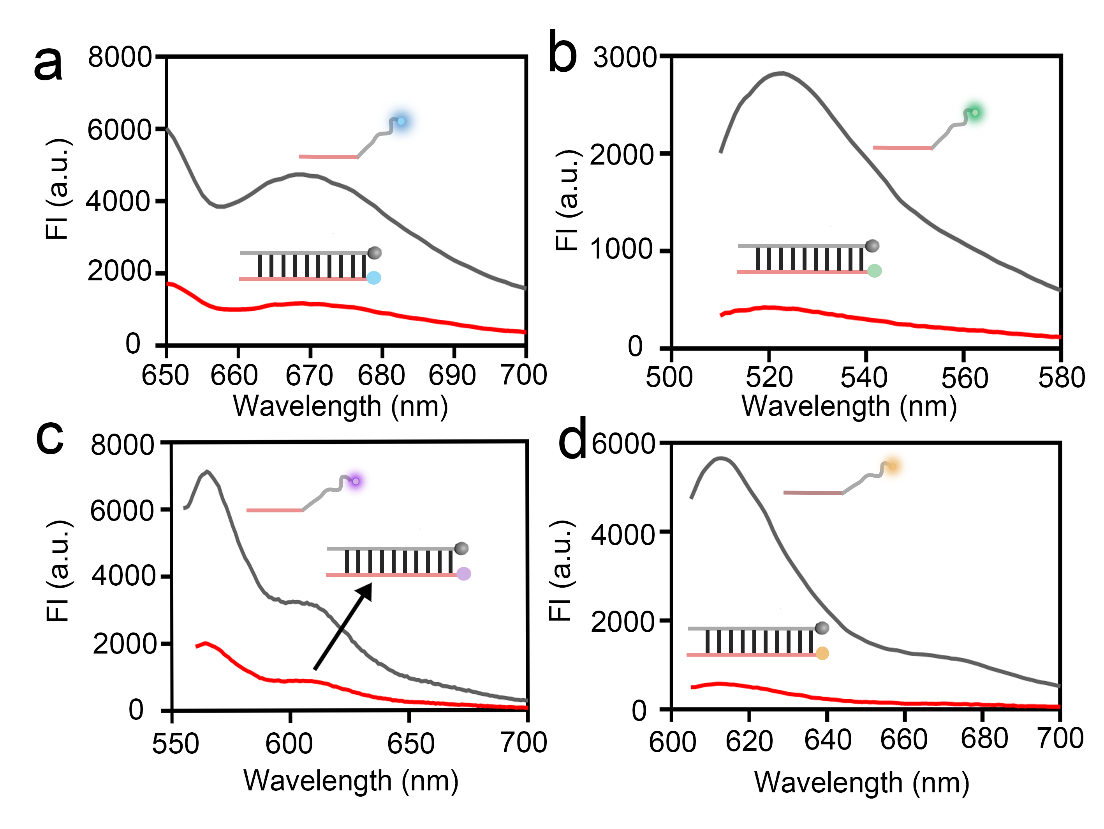


**Figure S6.** Fluorescence spectra of four different fluorophores, including (a) Cy5, (b) FAM, (c) Cy3, and (d) TR, in the presence or absence of BHQ2.


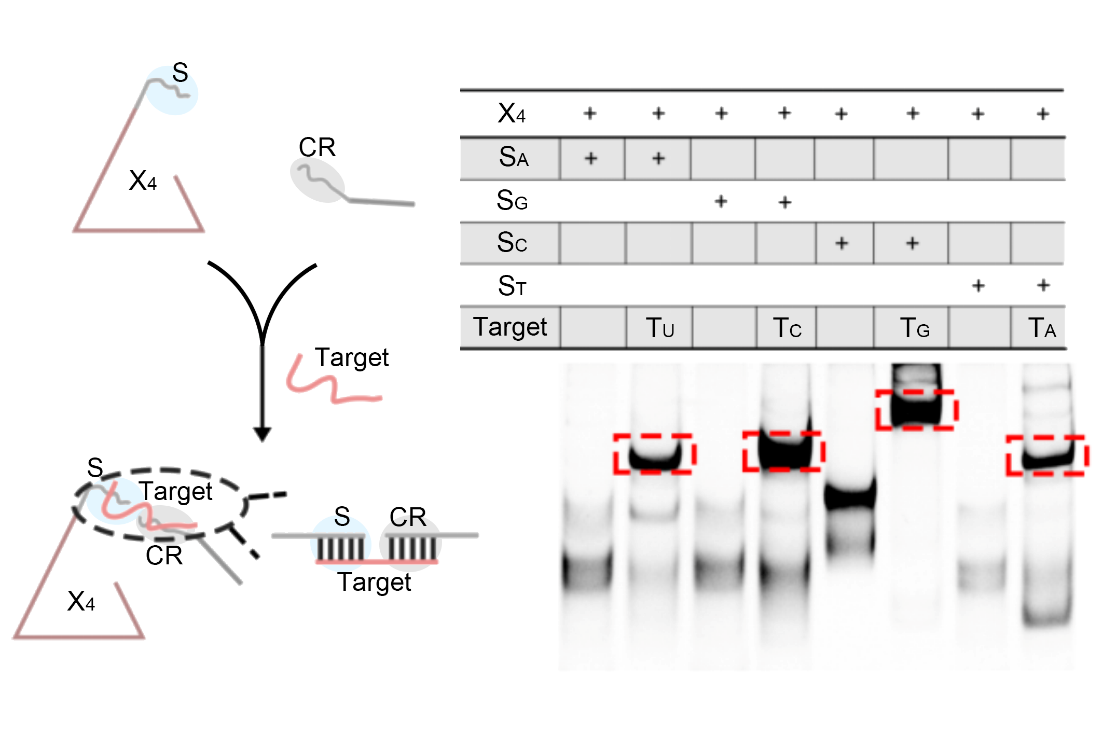


**Figure S7.** Polyacrylamide gel electrophoresis (PAGE) analysis to characterize the complementation of CR, S, and their corresponding target. The red boxes indicate the complementary products.


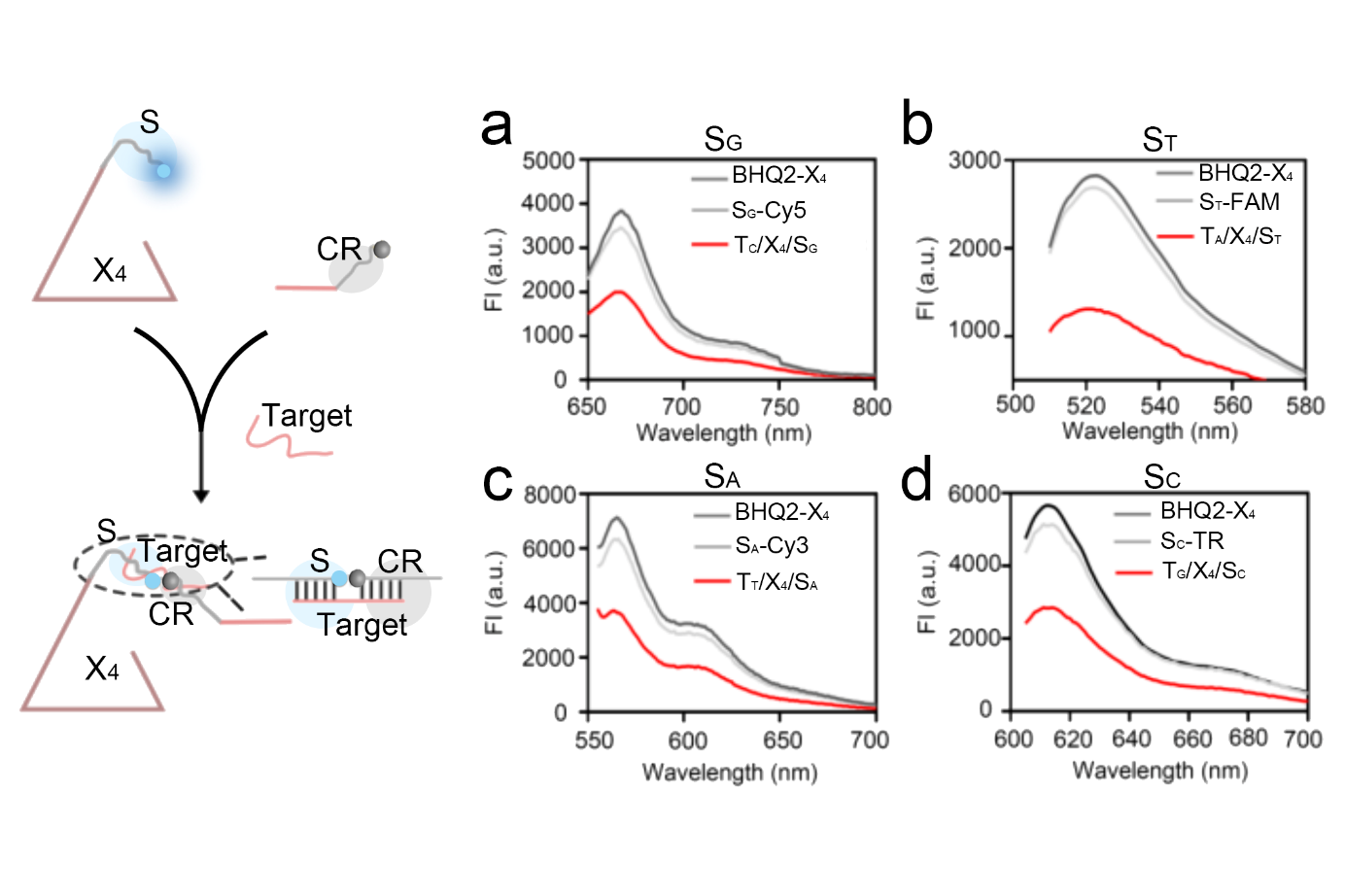


**Figure** **S8.**  Fluorescence intensity analysis of (a) Cy5-labeled S_G_, (b) FAM-labeled S_T_, (c) Cy3-labeled S_A_, and (d) TR-labeled S_C_ to determine the complementation of CR, S, and their corresponding target.


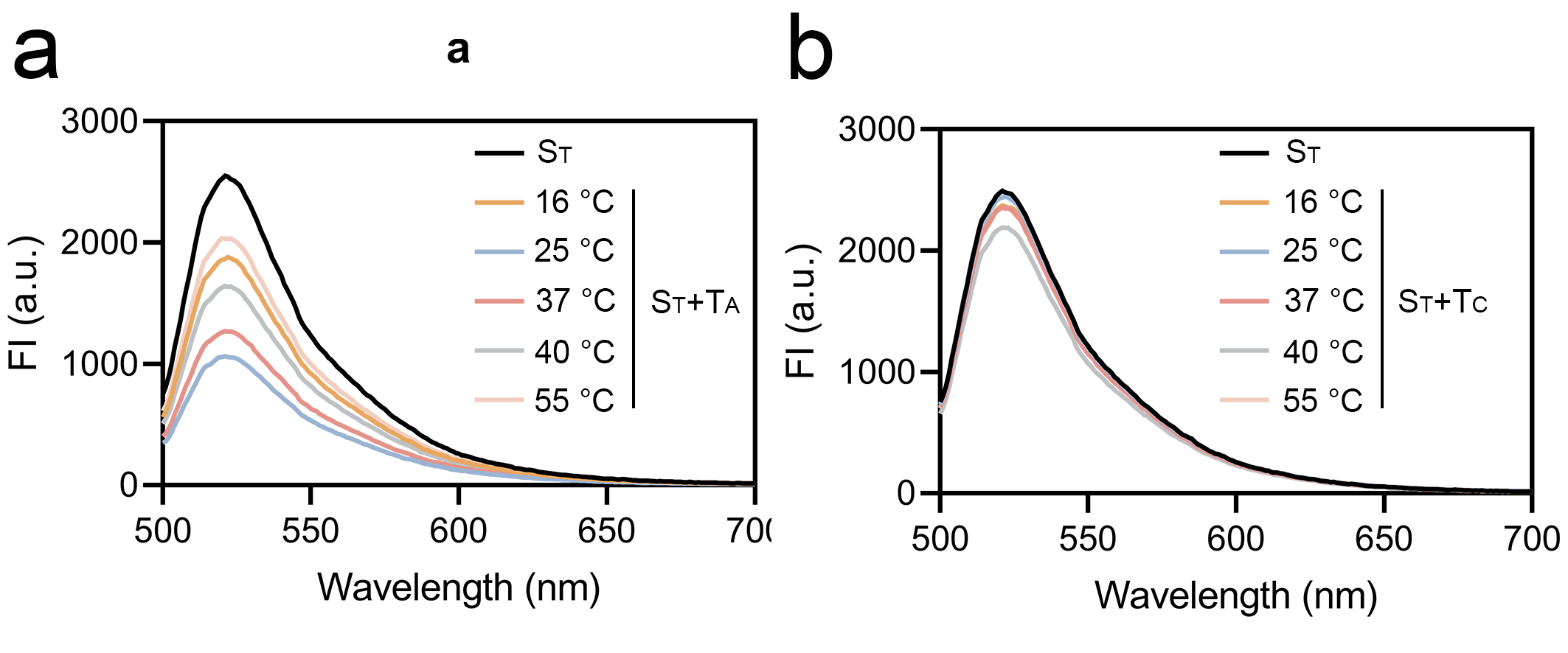


**Figure S9.** Fluorescence intensity analysis of FAM-labeled S_T_ after addition of the target T_A_ (a) or non-target T_C_ (b) at different temperatures.


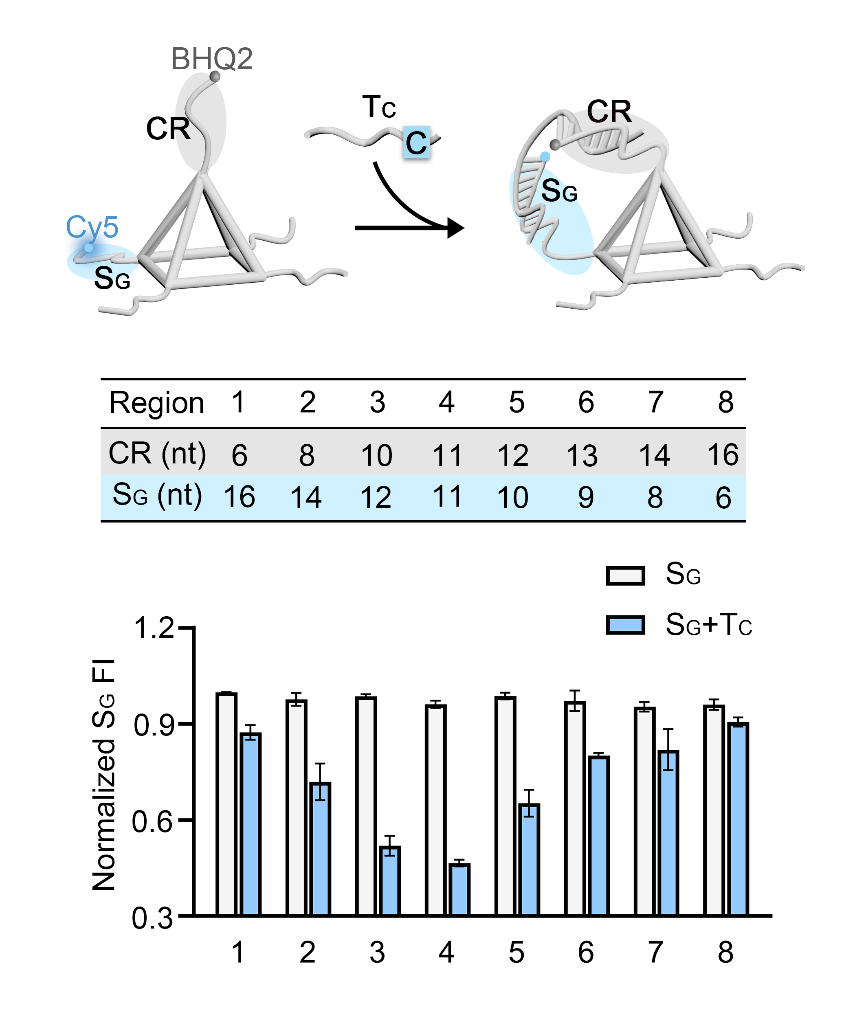


**Figure S10.** Normalized fluorescence intensity of Cy5-labeled S_G_ with different lengths of the CR and S regions after addition of the T_C_ for the optimization of recognizers. Data are the means ± SD, *n* = 3.


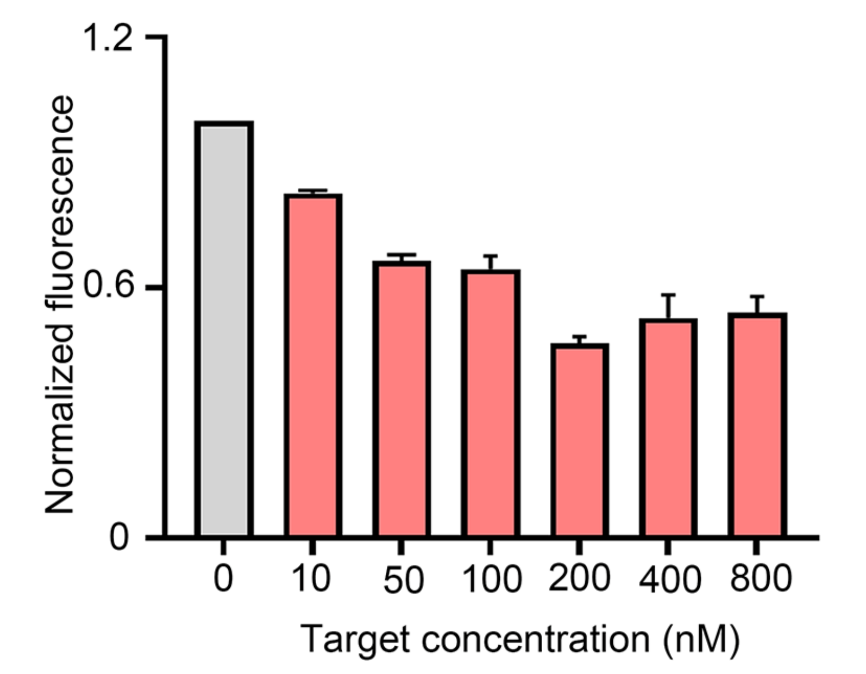


**Figure S11.** Normalized fluorescence intensity of Cy5-labeled S_G_ in response to different concentrations of T_C_. Data are the means ± SD, *n* = 3.


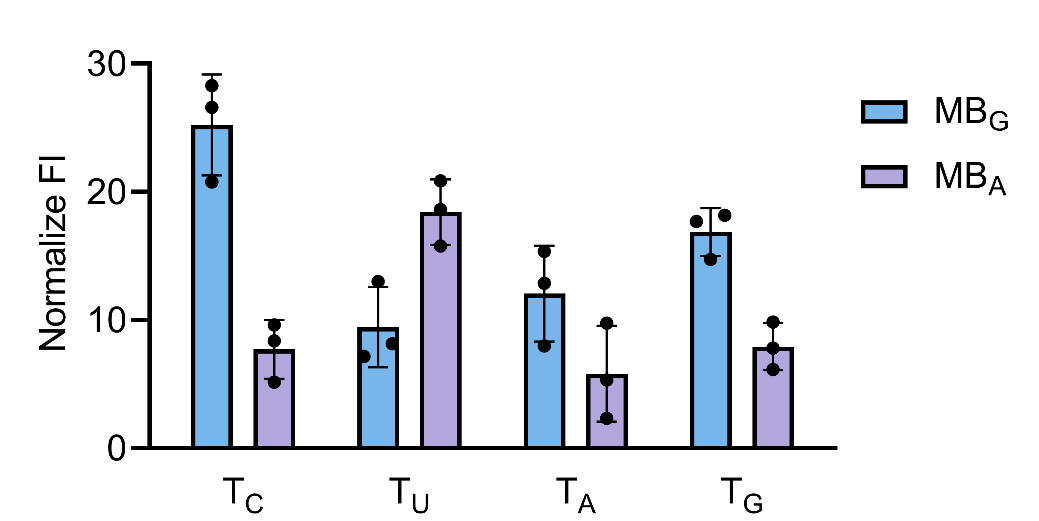


**Figure S12.** Normalized fluorescence intensity of MB_G_ and MB_A_ in MBs mixture after addition of the different miR-SNP targets, separately. Data are the means ± SD, *n* = 3.


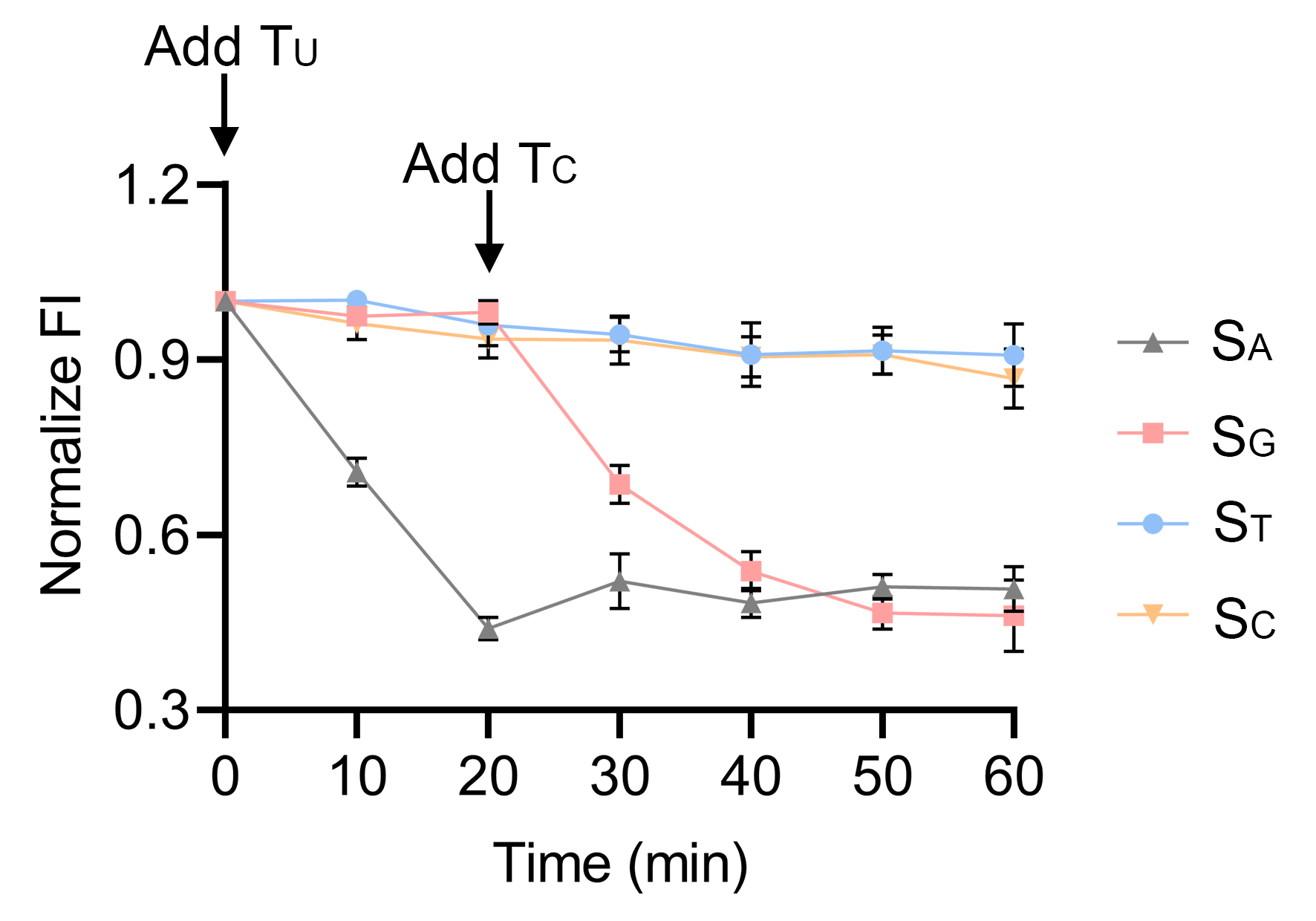


**Figure S13.** Dynamic normalized fluorescence intensity of the dynamic addressing process of DAMR after the addition of the different targets. Data are the means ± SD, *n* = 3.


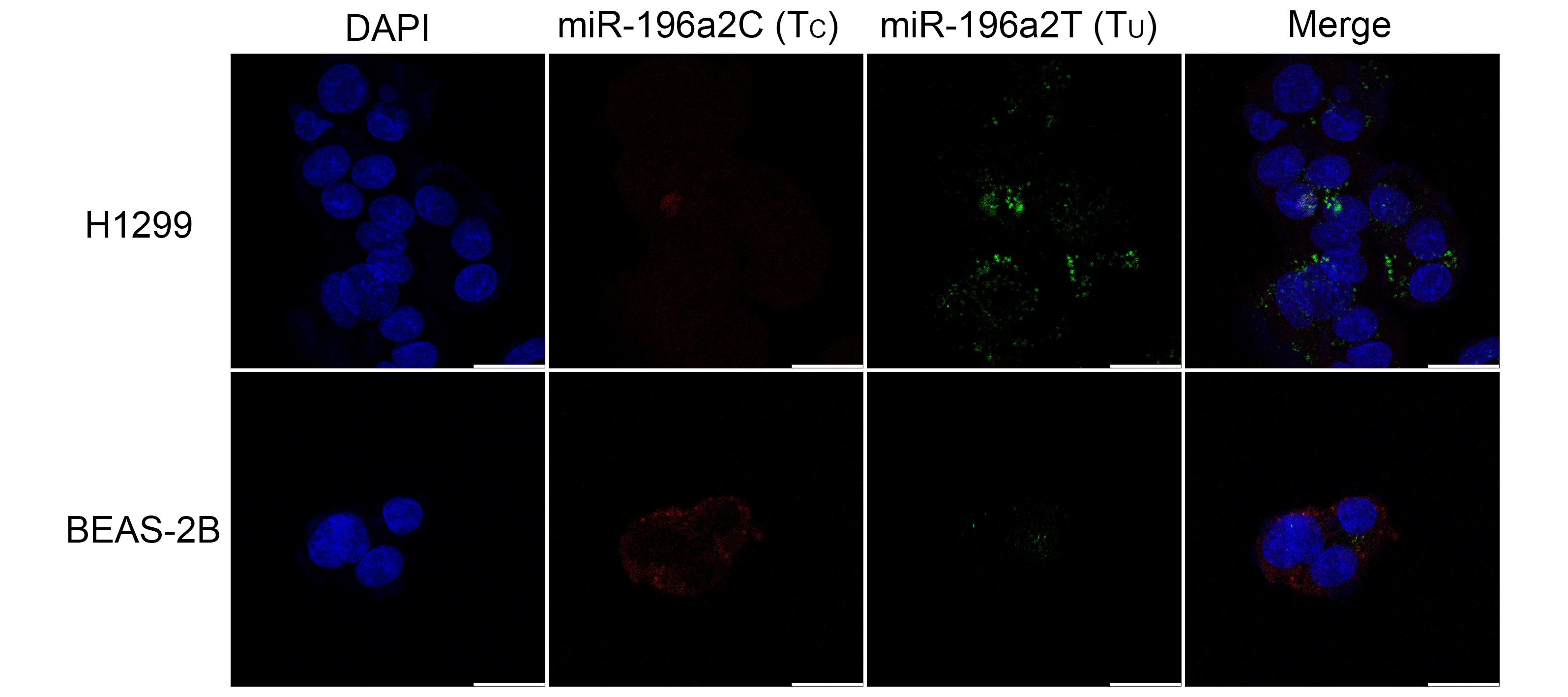


**Figure S14.** Fluorescence *in situ* hybridization (FISH) analysis of miR-196a2C and miR-196a2T in in H1299 and BEAS-2B cells. Scale bar = 20 μm.


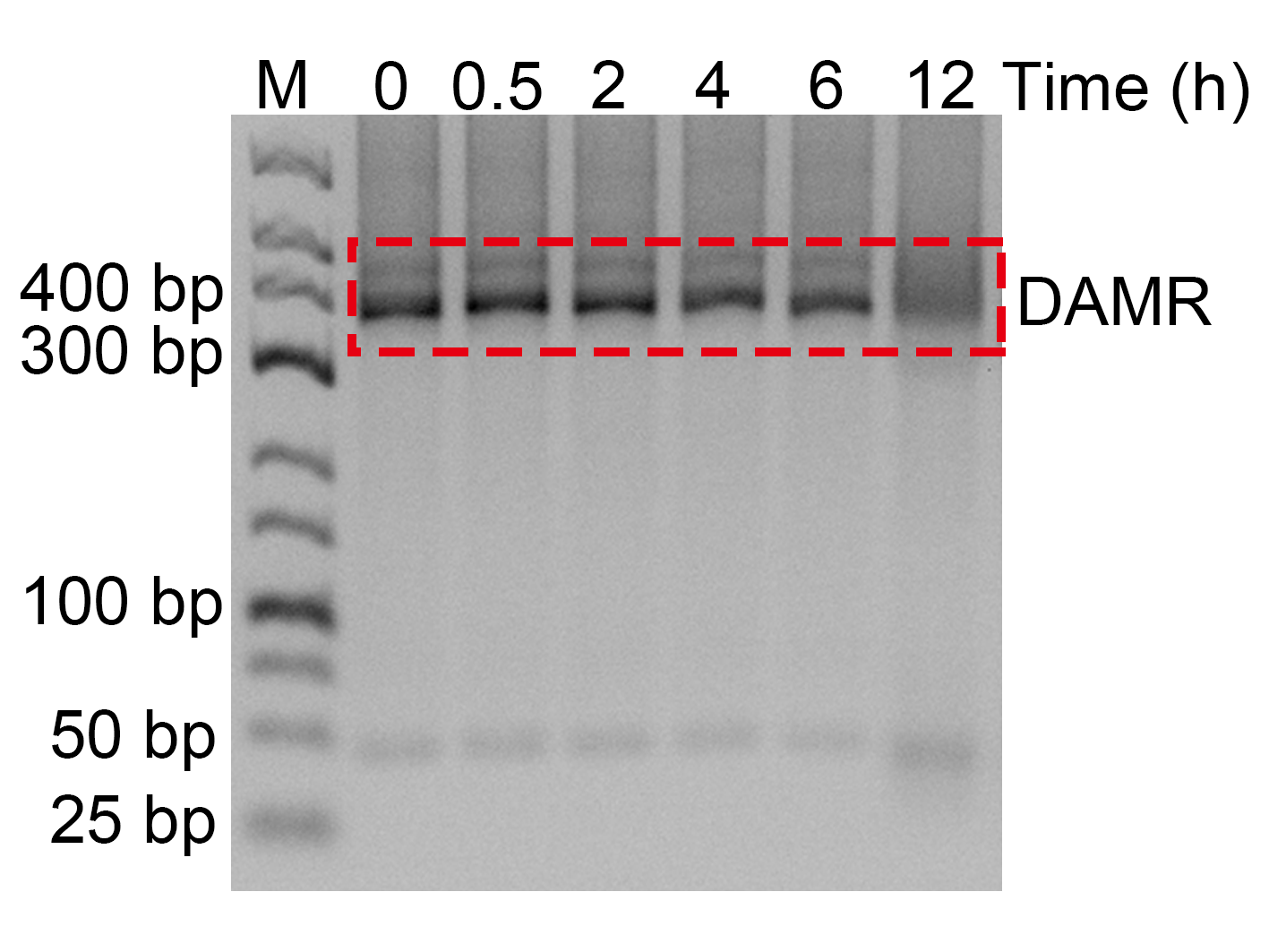


**Figure S15.** Agarose gel electrophoresis for investigating the stability of the DAMR in 10% serum from healthy individuals at room temperature for 0.5, 2, 4, 6, and 12 h.


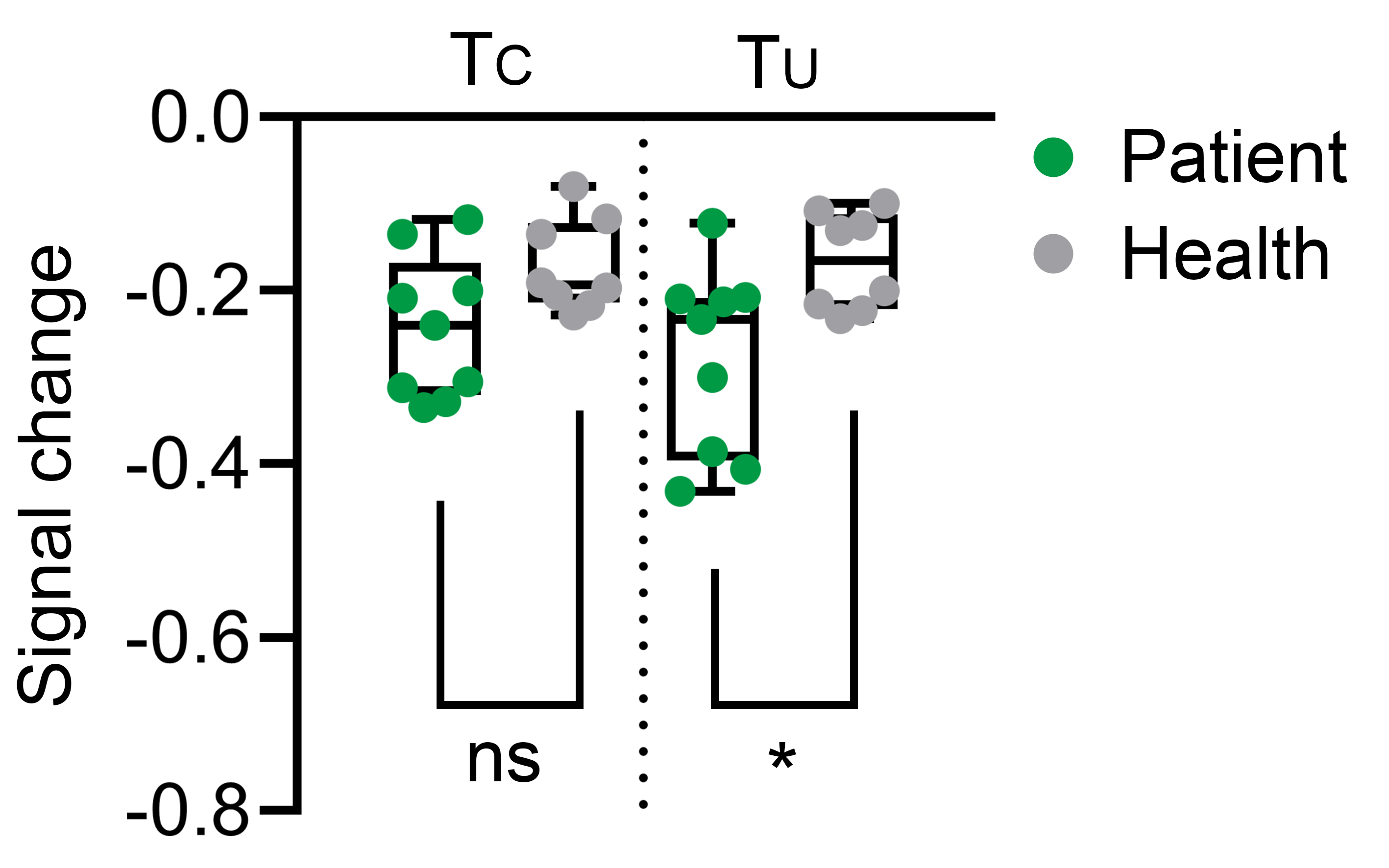


**Figure S16.** Signal changes of DAMR for analysis of miR-SNP targets in 5 μL of untreated serum samples from NSCLC patients (*n* = 9) and healthy individuals (*n* = 8). Data are the means ± SD, *p* values were calculated by the 2-tailed student's *t*-test, **p* < 0.1, ns means no significance.

**Table S1** Oligonucleotide sequences used in the study.

| Oligonucleotides | Sequence (5’-3’) |
| --- | --- |
| X_1_ | CGTTCACTAATTACGTTTTGAATCGATCCGTCTAGCCTTGCTGCTCTATTTTTATGATCAGGCTCAAGGGTTAACAGCACACGTTTTACACTCCTGAAGCTT |
| X_2_ | CTGCCATGTTCTCCATTTTCGTAATTAGTGAACGAAGCTTCAGGAGTGTTTTTATAAGGCGACGTAGCTGAGTGCGACTCGTCTTTTATCCTAAGACTGTGA |
| X_3_ | TAGACGGATCGATTCTTTTGTCGACCTGCCTACGCGTCAACGATCTAGTTTTTAGCGAACAGGACCGGAAGCGGTGCACGATGTTTTATAGAGCAGCAAGGC |
| X_4_ | TCTTGTTGCCGTTTTTTTTTTTTTTTTGGAGAACATGGCAGTCACAGTCTTAGGATTTTTCTGGAGGAGCTATGGCTGGAACTGCGTTGCTTTTACTAGATCGTTGACG-BHQ-2 |
| X_5_ | CGTAGGCAGGTCGAC |
| S_A_ | Cy3-CGTGTGCTGTTAACCCTTGAGCCTGATCATTTTTTTTTTTTTTTTCTCAG**A**CAGTT |
| S_G_ | Cy5-GACGAGTCGCACTCAGCTACGTCGCCTTATTTTTTTTTTTTTTTTCTCAG**G**CAGTT |
| S_C_ | TR-GCAACGCAGTTCCAGCCATAGCTCCTCCAGTTTTTTTTTTTTTTTCTCAG**C**CAGTT |
| S_T_ | FAM-CATCGTGCACCGCTTCCGGTCCTGTTCGCTTTTTTTTTTTTTTTTCTCAG**T**CAGTT |
| S_A_’ | AATGATCAGGCTCAAGGGTTAACAGCACACG-BHQ-2 |
| S_G_’ | AATAAGGCGACGTAGCTGAGTGCGACTCGTC-BHQ-2 |
| S_C_’ | ACTGGAGGAGCTATGGCTGGAACTGCGTTGC-BHQ-2 |
| S_T_’ | AAGCGAACAGGACCGGAAGCGGTGCACGATG-BHQ-2 |
| miR-196a2 C (T_C_) | CGGCAACAAGAAACUG***C***CUGAG |
| miR-196a2 T (T_U_) | CGGCAACAAGAAACUG***U***CUGAG |
| miR-196a2 G (T_G_) | CGGCAACAAGAAACUG***G***CUGAG |
| miR-196a2 A (T_A_) | CGGCAACAAGAAACUG***A***CUGAG |
| MB_A_ | BHQ2-CTCAGACAGTTTCTTGTTGCCGTTGGGGGTCTGAG-Cy3 |
| MB_G_ | BHQ2-CTCAGGCAGTTTCTTGTTGCCGTTGGGGGCCTGAG-Cy5 |
| MB_C_ | BHQ2-CTCAGCCAGTTTCTTGTTGCCGTTGGGGGGCTGAG-TR |
| MB_T_ | BHQ1-CTCAGTCAGTTTCTTGTTGCCGTTGGGGGACTGAG-FAM |
| FISH-miR-196a2C | CTCAGGCAGTTTCTTGTTGCCG-Cy3 |
| FISH-miR-196a2T | CTCAGACAGTTTCTTGTTGCCG-FAM |
